# Do-Not-Attempt-Resuscitation Orders in Older Adult Patients With Out-of-Hospital Cardiac Arrest: A Single-Center Retrospective Study at an Urban University Hospital Emergency Department in Japan

**DOI:** 10.7759/cureus.79552

**Published:** 2025-02-24

**Authors:** Mitsunobu Toyosaki, Koichi Ueno, Junichi Sasaki

**Affiliations:** 1 Department of Emergency and Critical Care Medicine, Keio University School of Medicine, Tokyo, JPN

**Keywords:** advance care planning (acp), do-not-resuscitate orders, end-of-life ethics, geriatric medicine, supportive and palliative care

## Abstract

Introduction

Despite its super-aging society, advance directives or physician orders for life-sustaining treatment are uncommon in Japan. This study aimed to determine the incidence of do-not-attempt-resuscitation (DNAR) orders in older adult patients with out-of-hospital cardiac arrest in Japan and whether cardiopulmonary resuscitation was canceled based on these orders.

Methods

We retrospectively analyzed patients with out-of-hospital cardiac arrest aged ≥65 years who were transported to and treated at Keio University Hospital emergency department between January 1, 2016, and December 31, 2018. Patients with in-hospital cardiac arrest, those treated by clinical departments other than the emergency department, and those transported from other hospitals were not included. We assessed the rate of DNAR orders and cardiopulmonary resuscitation terminations in patients in the emergency department with DNAR orders.

Results

Among the 142 included patients, 23 (16%) had not attempted resuscitation orders made by the patients or family members. Of these, six (26%) underwent continued cardiopulmonary resuscitation despite their DNAR orders. The reasons for continuing cardiopulmonary resuscitation were delays in confirming the DNAR orders and problems in understanding the DNAR orders. Patients of higher age, male sex, nursing home admission, and continued medical examination at our hospital were significantly more likely to desire DNAR orders.

Conclusions

In Japan, the number of DNAR orders among older adults is low. Furthermore, these orders are not sufficiently understood, even by medical workers. Thus, increased medical and social awareness of advance directives and physician orders for life-sustaining treatment is needed.

## Introduction

Advance directives (ADs) are legal documents used to indicate the types of treatments patients hope to receive in case of medical emergencies and are also a component of advanced care planning (ACP). ADs can identify surrogates in case patients cannot speak or indicate their intentions. In the United States, 72% of older adults (≥60 years of age) have completed ADs [1]. In a multicenter prospective study in Australia, approximately half of the older adults had some form of ACP (29.8% had ADs and 21.6% had other ACP documents) [2]. Studies not limited to the elderly in each country had shown the following AD rates; the United States (36.7% of adults aged ≥ 18 years) [3], Germany (2.5-10% of the general population) [4], the Netherlands (7% of adults aged ≥ 20 years) [5], the United Kingdom (8% of the general population) [6], Spain (0.05% of the general population) [7], and China (0% of cancer patients) [8]. There is considerable variation in subjects and data collection methods, and comparisons cannot be made easily, but it is thought that the level of understanding of AD varies considerably from country to country. While older adults in Japan appear to welcome information about ADs [9], approximately half of the Japanese palliative physicians do not recommend that patients complete an AD or do-not-attempt-resuscitation (DNAR) orders if they determine that the patient wishes for no cardiopulmonary resuscitation (CPR) in the event of cardiopulmonary arrest (CPA) [10]. All adults should ideally have ADs. However, ADs can be challenging to retrieve in medical emergencies and are only paper-based documents.

Physicians’ orders for life-sustaining treatment (POLSTs) are medical orders for the specific medical treatment required in medical emergencies. POLSTs are designed for individuals with serious illnesses or advanced frailty near the end of life. For instance, one can decide whether to receive CPR by implementing DNAR orders for CPA situations. Moreover, POLST forms are kept with a patient’s medical records; therefore, they can be easily located in medical emergencies, even by emergency medical services (EMS) [11].

In the United States, POLSTs are available at some levels, although the levels of recognition differ among states [11]. However, in Japan, POLSTs do not seem to be well understood by both the general public and medical professionals. There are no laws regarding POLSTs or EMS personnel not starting CPR on patients with DNAR orders. It is likely that POLSTs, like AD, are not well understood in Japan.

Regarding end-of-life care in the field of emergency and intensive care, a joint guideline was published in 2014 by three societies (the Japanese Association for Acute Medicine, the Japanese Society of Intensive Care Medicine, and the Japanese Circulation Society) [12]. According to these guidelines, if the patient has an AD, the principle is to respect that directive, but if there is a discrepancy with the family's wishes, discussion is required. It can be said that this showed a path forward in Japan. However, there is still little research on the extent to which ADs exist and whether they are actually respected. Furthermore, further consideration is needed regarding the difficulty of confirming ADs in the chaotic field of emergency medical care.

The present study focused on DNAR orders, as the simplest type of AD (or POLSTs), among older people (age ≥65 years). Moreover, in patients with predicted cardiac arrests, such as those with end-stage cancer in chronic hospitals, the rates of DNAR orders are expected to be high. Therefore, we selected situations of patients with out-of-hospital cardiac arrest (OHCA) who were transported to an acute hospital emergency department (ED). In this situation, the rates of DNAR orders may reflect one side of the current Japanese popularization of ADs in the older adult population. Whether CPR was canceled according to patient directives may allow a better view of the present situation of Japanese medical workers’ understanding and the maturities of the medical system. This study aimed to determine the incidence of DNAR orders in older adult patients with OHCA in Japan and whether CPR was canceled based on these DNAR orders.

## Materials and methods

This study was approved by the Institutional Review Board of the Keio University School of Medicine, Tokyo, Japan. A document clearly explaining the significance, objectives, methods, target period, and data to be collected (age, sex, current medical history, past medical history, family history, emergency transportation stage, presence or absence of nursing home admission, presence or absence of continued medical examination at Keio University Hospital, presence or absence of DNAR orders) was posted on the university's emergency department website.Informed consent was obtained from patients and their families who were eligible to be study subjects using an opt-out system.

Study design and setting

This retrospective single-center study included older adult patients with OHCA at the Keio University Hospital ED in Tokyo, Japan. Keio University Hospital is an urban hospital, with 7,000-8,000 patients transported to the ED annually.

Participants

The following patients were eligible for study inclusion: patients with OHCA aged ≥65 years transported to the ED at Keio University Hospital and treated by emergency physicians between January 1, 2016, and December 31, 2018. All causes of CPA (internal causes, external causes including trauma) were eligible for this study. Patients with in-hospital cardiac arrest, those treated by clinical departments other than the ED, and those transported from other hospitals were not included in this study.

Data collection and definitions

Data were collected regarding patient age, sex, primary waveform, CPR in the ED, ED disposition, transportation stage, nursing home admission, and continued medical examination at Keio University Hospital. ED disposition was defined as survival or death. Age groups were divided into early-stage seniors (<75 years of age) and late-stage seniors (≥75 years). The transportation stage was defined as normal or lifesaving. In Japan, patient transport to hospitals by emergency personnel is classified as a normal stage or lifesaving stage [13]. In patients with CPA, the normal transportation stage meant that the patients were not candidates for lifesaving measures, such as patients with DNAR orders or those judged obviously dead (e.g., those with rigor mortis) by the emergency personnel.

Our ED has no defined methods to confirm DNAR orders or CPR terminations. DNAR orders and CPR terminations were confirmed by ED faculty doctors and were recorded in the medical record system. DNAR orders were implemented according to the existence of DNAR documents or discussions the ED faculty doctors had with patients’ family members. Therefore, we checked our medical record systems and searched for the terms “DNAR confirmation” and “CPR termination.” In the case of “DNAR confirmation,” the type of DNAR orders (written or oral) and by whom (patients or family members) were collected. To simplify, we highlighted only DNAR orders; any other treatment requests (e.g., “do not intubate” or “do not use vasopressor”) were not collected. In prehospital settings in Japan, not starting CPR or CPR termination is decided by emergency medical technicians and is difficult owing to legal issues; therefore, the prehospital presence of CPR was not considered in this study.

Outcomes

The primary outcome was the rate of DNAR orders. The secondary outcome was the CPR termination rate in patients with DNAR orders in the ED. As an exploratory outcome measure, we analyzed factors related to DNAR orders.

Statistical analyses

Continuous variables were presented as means and standard deviations (SD), and categorical variables were presented as numbers and percentages. Statistical differences were compared using two-tailed Student’s t-tests for the difference in age and Fisher’s exact test for the difference in rates of male sex, nursing home admission, and continued medical examination at Keio University Hospital. Statistical significance was considered significant for p<0.05. All statistical analyses were conducted using Bellcurve for Excel (version 4.07; Social Survey Research Information Co., Ltd. Tokyo, Japan) and Microsoft Excel (Microsoft, Redmond, WA).

## Results

Characteristics

Of the 19,632 patients transported to and treated in the ED at Keio University Hospital between 2016 and 2018, 142 patients were enrolled in this study. No patients refused to participate in the study. In seven (4.9%) of the 142 patients, CPA was due to trauma or external causes. Two patients had experienced blunt trauma, one had choked on a rice cake, three were drowning, and one was an attempted suicide by hanging. The remaining 135 cases (95.1%) were assumed to be due to internal causes. No autopsy imaging or pathological autopsy was performed on any of the patients.

The patients’ average age was 80.44 years, and 51 (36%) were men. Moreover, 100 (70.4%) participants were late-stage seniors (Table 1).

**Table 1:** Participant characteristics Age is expressed as mean ± standard deviation (SD). Categorical variables are expressed as n (%). PEA, pulseless electrical activity; VF, ventricular fibrillation; VT, ventricular tachycardia; CPA, cardiopulmonary arrest; CPR, cardiopulmonary resuscitation; ED, emergency department

Characteristic	All patients (n=142)
Age (years)	80.44±8.45
65–74	40 (28.17)
75≦	102 (71.83)
Sex	
Male	51(35.92)
Female	91(64.08)
Primary waveform	
Asystole	99 (69.72)
PEA	27 (19.01)
VF/pulseless VT	6 (4.22)
Unknown	10 (7.04)
Estimated cause of CPA	
Internal	135 (95.07)
External	7 (4.93)
CPR in ED	
Continue CPR	114 (80.28)
Cancel CPR	28 (19.72)
ED disposition	
Survival	30 (21.13)
Death	112 (78.87)
Transportation stage	
Normal	27 (19.01)
Lifesaving	115 (80.99)
Nursing home admission	10 (7.04)
Continued medical examination at Keio University Hospital	19 (13.38)

Primary outcome

**Figure 1:**
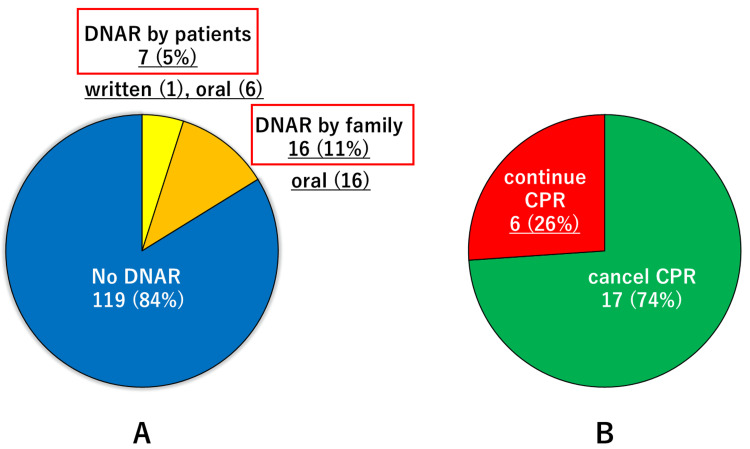
Rates of DNAR orders in older patients with OHCA and CPR cancel rates in patients with DNAR A: Rates of DNAR orders in older patients with OHCA. In the case of DNAR orders, the type of DNAR orders (written or oral) and by whom (patients or family members) were presented. B: CPR cancel rates in patients with DNAR. The prehospital presence of CPR was not considered. DNAR, do-not-attempt resuscitation; OHCA, out-of-hospital cardiac arrest; CPR, cardiopulmonary resuscitation

Twenty-three patients (16%) had DNAR orders, while 119 patients (84%) did not (Figure 1A). Among the patients with DNAR orders, only one had written DNAR orders. Six patients had only oral DNAR orders, or 16 DNAR orders made by their family members. Only seven patients made the DNAR orders themselves.

All patients with DNAR orders were late-stage seniors, except one patient with end-stage cancer. In other words, no “healthy” early-stage seniors had DNAR orders.

Secondary outcome

Table 2 presents the characteristics of the 23 patients with DNAR orders.

**Table 2:** Characteristics of all patients with DNAR orders DNAR, do-not-attempt resuscitation; CPA, cardiopulmonary arrest; CPR, cardiopulmonary resuscitation; ED, emergency department; PEA, pulseless electrical activity

Age	Sex	Primary wave form	Estimated cause of CPA	ED disposition	DNAR	CPR in ED	Transportation stage	Reason for continuing CPR in ED	Nursing home admission	Continued medical examination at Keio University Hospital
89	F	Asystole	Internal	Death	Patient	Yes	Lifesaving	Misunderstanding of DNAR	Yes	No
88	F	Asystole	Internal	Death	Family members	Yes	Lifesaving	Initially cannot confirm DNAR	No	Yes
83	M	Asystole	Internal	Death	Family members	Yes	Normal	“Partial” DNAR	No	Yes
91	M	Asystole	Internal	Survival	Patient	Yes	Lifesaving	Initially cannot confirm DNAR	No	No
89	M	Asystole	Internal	Death	Patient	Yes	Lifesaving	Initially cannot confirm DNAR	No	No
78	F	Asystole	Internal	Survival	Patient	Yes	Lifesaving	Family members wished for CPR	No	No
91	F	Asystole	Internal	Death	Family members	No	Normal	-	No	No
100	M	Asystole	Internal	Death	Family members	No	Normal	-	Yes	No
74	F	Asystole	Internal	Death	Patient	No	Normal	-	No	Yes
80	M	Asystole	Internal	Death	Patient	No	Normal	-	No	Yes
81	M	Asystole	Internal	Death	Family members	No	Normal	-	No	No
88	M	Asystole	Internal	Death	Family members	No	Normal	-	No	No
93	M	Asystole	Internal	Death	Family members	No	Normal	-	Yes	No
84	M	Unknown	Internal	Death	Family members	No	Normal	-	No	No
89	F	Unknown	Internal	Death	Patient	No	Normal	-	No	Yes
94	F	Asystole	Internal	Death	Family members	No	Normal	-	No	No
94	F	Asystole	Internal	Death	Family members	No	Normal	-	No	No
81	M	Asystole	Internal	Death	Family members	No	Normal	-	No	Yes
89	F	Asystole	Internal	Death	Family members	No	Normal	-	No	No
83	F	Asystole	Internal	Death	Family members	No	Normal	-	No	No
95	F	PEA	Internal	Death	Family members	No	Normal	-	Yes	No
79	F	Asystole	Internal	Death	Patient	No	Normal	-	Yes	Yes
90	F	Unknown	Internal	Death	Family members	No	Normal	-	No	No

Naturally, patients with no DNAR orders received CPR, although six patients with DNAR orders (26%) received CPR contrary to their wishes (Figure 1B). In half of the patients, CPR was continued because of a failure to confirm the DNAR orders.

Factors related to DNAR orders

Patient factors related to DNAR orders (age, sex, nursing home admission, and continued medical examination at Keio University Hospital) are presented in Table 3. Patients with older age (p<0.001), male sex (p=0.033), nursing home admission (p=0.011), and undergoing continued medical examination at Keio University Hospital (p=0.016) were significantly more likely to desire DNAR orders.

**Table 3:** Comparison of age, sex, nursing home admission, and continued medical examination at our hospital among patients with and without a DNAR order Age is expressed as mean ± standard deviation (SD). Categorical variables are expressed as n (%). DNAR, do-not-attempt resuscitation

Characteristics	All patients (n=142)	DNAR (+) (n=23)	DNAR (-) (n=119)	P-value
Age, mean years±standard deviation (range)	80.44±8.45 (66–100)	87.09±6.26 (74–100)	79.15±8.21 (66–97)	<0.001
Male sex, n (%)	51 (36)	13 (57)	38 (32)	0.033
Nursing home admission, n (%)	10 (7.0)	5 (22)	5 (4.2)	0.011
Continued medical examination at Keio University Hospital, n (%)	19 (13)	7 (30)	12 (10)	0.016

## Discussion

Enlightenment regarding ADs and POLSTs

The rates of DNAR orders (16%) in our study are low compared to the AD completion rates in the United States [1]. At the same time, they are not significantly different from a previous prehospital study in Japan, in which 11.4% of patients had DNAR orders (80.6% of patients were aged ≥65 years) [14]. In Japan, older adults have few opportunities to learn about ADs and POLSTs. Consequently, older adults and their family members are unsure whether to undergo CPR in CPA situations. A Japanese version of POLSTs [15] exists, although few medical institutions use it. Moreover, it is not easily accessed by paramedics from outside medical institutions. Therefore, it is unsuitable for use in emergencies. POLST is required in seriously ill or frail patients. Although the definitions of frail patients differ, most super-old (≥90 years) people are considered frail. In our study, 21 patients (15%) were super-old, and eight had DNAR orders, although only one patient had made these orders himself.

Legal problems

Caring for end-of-life patients has legal challenges. Unfortunately, many legal myths exist regarding end-of-life care, even in the United States [16]. Moreover, laws regarding ADs and POLSTs are yet undeveloped in Japan. In many prehospital situations, paramedics cannot refrain from starting (and also cannot terminate) CPR on patients with DNAR orders in Japan [14]. Complex conditions are required to avoid initiating CPR in prehospital settings. For example, the Tokyo Fire Department will not attempt CPR in the following pre-hospital settings: 1) adults in CPA with pre-existing ACP; 2) patients facing the end stages of their life; 3) the patients desire no CPR; and 4) situations in which patients are assumed in advance to match the current situation. Moreover, confirmation by family doctors is required [17]. If even one condition is unsatisfied, patients may receive undesired CPR and transport to emergency departments. Although these situations should be improved, Japanese paramedics are restricted in many respects, with limited authority. Therefore, improvements in the prehospital CPA situations of patients with DNAR are challenging until legal guidelines surrounding Japanese paramedics are revised.

Accuracy of surrogate decision-making

In our study, many (70%) DNAR orders were made by family members or surrogates and not by the patients, which raises problems with accuracy. In one systematic review, surrogates predicted patients’ treatment preferences with 68% accuracy and 69% accuracy for CPR [18]. Moreover, patients’ designation of surrogates, surrogates’ relationships with the patients [19-22], and discussions of patients’ treatment preferences [23,24] with surrogates are ineffective in improving surrogates’ decision-making accuracy. Therefore, blindly following surrogate decisions may not always be appropriate. Furthermore, it is important to be aware that Japanese physicians already tend to prefer family-centered decision-making [10]. Some surrogates use phrases such as “We believe that dad (or mum) wished no CPR if he (or she) could talk.” However, confirmation of the accuracy of surrogate decision-making in CPR situations is difficult.

Problems in the timing of decision-making: was the DNAR order decided in advance?

Some DNAR orders made by family members were considered “post-directives.” In the prehospital setting, paramedics often ask simple questions to family members who are not on the scene, such as “Your parent is now in cardiac arrest. Do you wish for resuscitation to be performed?” at which time the family members make immediate decisions, and these family members (and the patients themselves) have not thought of an AD or POLST in advance. At least two DNAR orders were confirmed by paramedics on the phone. Details of prehospital confirmations of these two cases were unknown from medical record systems. Therefore, this was an important limitation of this study and raised difficulties in the confirmation of DNAR as an AD.

Undesired CPR in patients with DNAR orders

Undesired CPR was implemented even in hospitals. Six patients underwent undesired CPR, three owing to problems confirming DNAR orders and the others owing to problems understanding DNAR orders. In Japan, many older adults only make DNAR orders orally, and few family members are aware of these orders. In emergency situations (e.g., sudden CPA), oral-only DNAR orders are difficult to confirm by other family members, much less by paramedics.

Among the reasons for a lack of understanding, “misunderstanding of DNAR among family members” indicates a lack of uniformity in understanding the patient’s living will among family members. Some family members reported that the patient wished for no suffering at the end of life; however, other members disagreed. “Family members wished for CPR” also reflects a lack of uniformity in understanding the patient’s living will. In one case, the patient wished for no CPR, whereas her husband wished for full CPR. The patient was diagnosed with end-stage breast cancer. As mentioned previously, surrogate decision-making problems underlie these two cases. “Partial” DNAR is a challenge for health professionals. In this DNAR order, intubation and mechanical ventilation were undesired, although other resuscitation actions, such as chest compressions and epinephrine injections, were desired. Unfortunately, the patient gained temporary recovery of spontaneous circulation before experiencing CPA and finally being declared dead.

Transportation stage

Focusing on the transportation stage, 27 (19%) of the 142 cases were normal transportation. The reasons for normal transportation were as follows: 18 had DNAR orders, four were assessed by paramedics not to be candidates for lifesaving, four were transported to our hospital from far away according to their families’ wishes, and one was due to mistakes in confirmation of DNAR orders.

Of the 18 normal transportation patients with DNAR orders, 17 patients, except for one patient with a “partial” DNAR, as mentioned above, had CPR canceled following their wishes. This means that, if the DNAR orders had already been confirmed by emergency medical personnel, it would not have been difficult for the hospital to follow the DNAR orders. This suggests that early confirmation is important to ensure that a patient’s DNAR orders are followed. Additionally, all four patients transported according to their families’ wishes underwent continued examination at our hospital, and the family desired them to be transported to our hospital, even if it was far away. One patient with mistakes in the confirmation of DNAR orders was confirmed to have no DNAR orders after transportation. The medical record did not provide a reason.

Factors related to DNAR orders

The results of the present study revealed four significant factors related to DNAR:

Age: It is naturally understood as an important reason for avoiding futile care (e.g., CPR) by both patients and family members.

The higher wish for DNAR for “male sex” contradicts previous study findings: One study on adult patients with cancer reported a higher desire for resuscitation and dialysis in men than in women [25]. Moreover, another study showed that male physicians and physicians working in clinics were less likely to agree that ACP should be provided by medical care staff [26]. These findings suggest that male patients and physicians are passive regarding ACP. This contradiction cannot be explained easily. Patients in our study were not healthy; therefore, heterogeneity might explain this difference.

Nursing home admission: It is a key factor when considering ACP in patients and their families. In the United States, nursing homes were the place of death for approximately 20-25% of older adults (age ≥65 years) from 2018-2022 [27]. Similarly, in Japan, 16% of older adults died in nursing homes in 2022 [28]. Nursing homes are expected to provide end-of-life care for older adults. Upon admission, most nursing homes ask patients and their families for direction regarding ACP. Most patients in nursing homes desired DNAR orders, as they wished to spend their final days without pain or suffering [26].

Continued medical examinations at our hospital: It is also a key factor in ACP. Older adults continuing medical treatment may have more opportunities to consider their lives and ACP than those without medical treatment. Although the difference was not significant, advance directive completion was higher among patients (38.2%) than among healthy adults (32.7%) in one US study [3]. Further, “at our hospital” is an important consideration while confirming patients’ wishes. As mentioned above, it is difficult to confirm patients’ wishes during emergencies. However, the medical records at their own hospital can be easily confirmed.

Possible future improvements

In summary, ACP is uncommon among older Japanese people. Although DNAR orders are only one aspect of ACP, they are easily understood by older adults. It is believed that this issue can be improved in the future if medical professionals and the government actively provide information to the elderly and provide them with opportunities to make choices. Specifically, one method that could be considered would be to provide a simple option for advance directives on health insurance cards. It is also possible that religious and cultural aspects unique to Japan may also have had an influence, and detailed research into these aspects is needed.

We pointed out the need for improvement in Japan's medical system, which leads to undesired CPR. This is a problem that is difficult to correct immediately, including legal problems. Nevertheless, the strength of our study is that we were able to point out the problem with concrete figures. Undesired CPR can be prevented by adjusting prehospital systems and systematizing POLSTs between family hospitals and local emergency medical systems. Finally, we identified four elements linked to DNAR orders.

Limitations

The limitations of our study include the single-center design of the urban university hospital ED and some selection biases. The data collection period was before the coronavirus disease 2019 (COVID-19) pandemic; therefore, our data do not reflect the latest situations post-COVID-19. At the same time, some important points related to DNAR orders could not be collected because of the retrospective study design based on medical records. Information of pre-existing ACP other than DNAR and activities of daily living (ADL) are representative. The presence of ACP could be confirmed only in some terminally ill patients with continued examination at Keio University Hospital, although, in most other patients, ACP information was missing and unknown. Similarly, ADL information (e.g., independent or bedridden) could not be confirmed sufficiently in most patients. Therefore, the possibility that these factors influenced DNAR orders as confounding factors could not be ruled out. Additionally, the study population included only patients with OHCA, and other patients transported to the ED with severe statuses other than CPA were not included. The medical background of our population was not uniform. Because of these limitations, our findings cannot be generalized easily. However, our study shows one perspective on the current situation of DNAR orders in older adult Japanese patients. Multicenter prospective studies are needed in the future.

## Conclusions

The rate of DNAR orders in older adult patients with OHCA in Japan, a super-aging society, is low. In older Japanese patients, DNAR orders are insufficiently understood. Awareness regarding ACP among older adults and their family members is required.

Additionally, undesirable CPR was occasionally implemented. Like POLSTs in the United States, systems are needed in Japan to ensure timely confirmation of DNAR orders in prehospital situations. Early confirmation is important to ensure adherence to patients’ DNAR orders. It is also advisable for patients to prepare in advance so that their advance directives can be easily verified by others, including family members, in the event of an emergency.

## References

[1] Silveira MJ, Wiitala W, Piette J (2014). Advance directive completion by elderly Americans: a decade of change. J Am Geriatr Soc.

[2] Detering KM, Buck K, Ruseckaite R (2019). Prevalence and correlates of advance care directives among older Australians accessing health and residential aged care services: multicentre audit study. BMJ Open.

[3] Yadav KN, Gabler NB, Cooney E (2017). Approximately one in three US adults completes any type of advance directive for end-of-life care. Health Aff (Millwood).

[4] Evans N, Bausewein C, Meñaca A (2012). A critical review of advance directives in Germany: attitudes, use and healthcare professionals' compliance. Patient Educ Couns.

[5] van Wijmen MP, Rurup ML, Pasman HR, Kaspers PJ, Onwuteaka-Philipsen BD (2010). Advance directives in the Netherlands: an empirical contribution to the exploration of a cross-cultural perspective on advance directives. Bioethics.

[6] Aw D, Hayhoe B, Smajdor A, Bowker LK, Conroy SP, Myint PK (2012). Advance care planning and the older patient. QJM.

[7] Simon-Lorda P, Tamayo-Velázquez MI, Barrio-Cantalejo IM (2008). Advance directives in Spain. Perspectives from a medical bioethicist approach. Bioethics.

[8] Zheng RJ, Fu Y, Xiang QF (2016). Knowledge, attitudes, and influencing factors of cancer patients toward approving advance directives in China. Support Care Cancer.

[9] Miki R, Becker CB, Ide K, Kawakami K (2018). Timing and facilitation of advanced directives in Japan. Arch Gerontol Geriatr.

[10] Nakazawa K, Kizawa Y, Maeno T, Takayashiki A, Abe Y, Hamano J, Maeno T (2014). Palliative care physicians' practices and attitudes regarding advance care planning in palliative care units in Japan: a nationwide survey. Am J Hosp Palliat Care.

[11] (2025). POLST basics. https://polst.org/about/.

[12] (2025). [Guidelines for end-of-life care in emergency and intensive care]. https://www.jsicm.org/pdf/1guidelines1410.pdf.

[13] (2025). [Urgency assessment protocol ver. 3: emergency scene]. https://www.fdma.go.jp/mission/enrichment/appropriate/items/kyukyu.pdf.

[14] Maruhashi T, Oi M, Asakuma S, Kotoh R, Shibuya H, Kurihara Y, Asari Y (2021). Advanced do-not-attempt-resuscitation directives and emergency medical services for out-of-hospital cardiopulmonary arrest patients in Japan: a pilot study. Acute Med Surg.

[15] (2025). [Creation guidelines of Japanese POLST (including DNAR)]. https://c-ethics.jp/deliverables/detail02/.

[16] Meisel A, Snyder L, Quill T (2000). Seven legal barriers to end-of-life care: myths, realities, and grains of truth. JAMA.

[17] (2025). [How to deal with injured or ill people who do not want CPR]. https://www.tfd.metro.tokyo.lg.jp/lfe/kyuu-adv/acp.html.

[18] Shalowitz DI, Garrett-Mayer E, Wendler D (2006). The accuracy of surrogate decision makers: a systematic review. Arch Intern Med.

[19] Suhl J, Simons P, Reedy T, Garrick T (1994). Myth of substituted judgment. Surrogate decision making regarding life support is unreliable. Arch Intern Med.

[20] Sulmasy DP, Haller K, Terry PB (1994). More talk, less paper: predicting the accuracy of substituted judgments. Am J Med.

[21] Sulmasy DP, Terry PB, Weisman CS, Miller DJ, Stallings RY, Vettese MA, Haller KB (1998). The accuracy of substituted judgments in patients with terminal diagnoses. Ann Intern Med.

[22] Tomlinson T, Howe K, Notman M, Rossmiller D (1990). An empirical study of proxy consent for elderly persons. Gerontologist.

[23] Ditto PH, Danks JH, Smucker WD (2001). Advance directives as acts of communication: a randomized controlled trial. Arch Intern Med.

[24] Matheis-Kraft C, Roberto KA (1997). Influence of a values discussion on congruence between elderly women and their families on critical health care decisions. J Women Aging.

[25] Golombek T, Hegewald N, Schnabel A, Fries H, Lordick F (2024). Stability of end-of-life care wishes and gender-specific characteristics of outpatients with advanced cancer under palliative therapy: a prospective observational study. Oncol Res Treat.

[26] Sakamoto A, Inokuchi R, Iwagami M, Hanari K, Tamiya N (2023). Association between physicians' characteristics and their knowledge, attitudes, and practices regarding advance care planning: a cross-sectional study. BMC Palliat Care.

[27] (2025). Multiple cause of death, 2018-2023, single race request. https://wonder.cdc.gov/mcd-icd10-expanded.html.

[28] (2025). [Statistical table and graph display]. https://www.e-stat.go.jp/dbview?sid=0003411690.

